# Limited Memory-Based Random-Weighted Kalman Filter

**DOI:** 10.3390/s24123850

**Published:** 2024-06-14

**Authors:** Zhaohui Gao, Hua Zong, Yongmin Zhong, Guangle Gao

**Affiliations:** 1 School of Electronic Engineering, Xi’an Shiyou University, Xi’an 710065, China; 2 National Key Laboratory of Science and Technology on Aerospace Intelligent Control, Beijing Aerospace Automatic Control Institute, Beijing 100854, China; zonghua3@sina.cn; 3 School of Engineering, RMIT University, Bundoora, Melbourne 3083, Australia; 4 School of Automatics, Northwestern Polytechnical University, Xi’an 710072, China; gaole1211@163.com

**Keywords:** Kalman filter, limited memory, random weighting estimation, noise statistics, unbiased estimation

## Abstract

The Kalman filter is an important technique for system state estimation. It requires the exact knowledge of system noise statistics to achieve optimal state estimation. However, in practice, this knowledge is often unknown or inaccurate due to uncertainties and disturbances involved in the dynamic environment, leading to degraded or even divergent filtering solutions. To address this issue, this paper presents a new method by combining the random weighting concept with the limited memory technique to accurately estimate system noise statistics. To avoid the influence of excessive historical information on state estimation, random weighting theories are established based on the limited memory technique to estimate both process noise and measurement noise statistics within a limited memory. Subsequently, the estimated system noise statistics are fed back into the Kalman filtering process for system state estimation. The proposed method improves the Kalman filtering accuracy by adaptively adjusting the weights of system noise statistics within a limited memory to suppress the interference of system noise on system state estimation. Simulations and experiments as well as comparison analysis were conducted, demonstrating that the proposed method can overcome the disadvantage of the traditional limited memory filter, leading to im-proved accuracy for system state estimation.

## 1. Introduction

The Kalman filter (KF) is widely used in many fields such as guidance and navigation, biological tissue characterization, and power systems [1,2,3]. Under the condition that system noise statistics are pre-defined accurately, KF can produce accurate state estimation results [4,5,6]. However, it needs to know the accurate statistical characteristics of system noise. In engineering practice, such as global positioning systems and inertial navigation integrated systems, due to the uncertainties and interferences involved in the dynamic environment, the statistical characteristics of system noise are usually unknown or inaccurate, which leads to biased or even divergent KF solutions [7,8,9]. Thus, a robust filtering technique is needed to overcome the influence of unknown or inaccurate noise statistics on the system.

In this paper, by combining the concepts of random weighting and limited memory technology into KF, a random-weighted Kalman filter (LM-RWKF) based on limited memory is developed to achieve the accuracy and reliability of system state estimation under unknown or inaccurate noise statistics. This method estimates system noise statistics by adaptive adjusting the weights of noise statistics within a limited memory to restrain the interferences of system noise and excessive historical information on system state estimation. Based on the limited memory technique, random weighting theories are established to estimate and adjust both process noise and measurement noise statistics within a limited memory via random weights. The estimated system noise statistics are further fed back into the Kalman filtering process for system state estimation. Simulations and experiments, as well as comparison analysis with the proposed LM–RWKF and the traditional limited memory KF (LM–KF), were conducted to comprehensively evaluate the LM-RWKF performance.

## 2. Related Work

To achieve accuracy and reliability of system state estimation under unknown or inaccurate noise statistics, various techniques of noise statistics estimation have been reported for KF. The correlation method estimates system noises statistics based on the innovation sequence. However, it is not optimal in terms of mean square error [10,11]. The Sage–Husa adaptive filtering improves the filtering accuracy by adaptively adjusting system noise covariances [12,13,14,15]. However, since system noise covariances are estimated by arithmetic mean, it may not guarantee that the final filtering solution is optimal. As an approximation to Bayesian estimation, multiple-model based robust KF tracks time-varying noise statistics by simultaneously operating a set of KFs. However, this method suffers from substantial computational complexity [16,17,18,19]. Fuzzy logic estimates system noise statistics to improve the KF adaptability and robustness [20]. However, since fuzzy rules are developed based on empiricism and heuristic information, the resultant estimation has limited performance. Fault detection is also used to handle inaccurate system noise statistics [21,22]. However, this technique is only able to identify and isolate the occurrence of inaccurate system noise statistics, while being incapable of suppressing their influence on state estimation. The maximum likelihood theory estimates noise statistics through the maximization of their posteriori probability density [23,24]. However, due to the involvement of too many historical residuals, the resultant estimations cannot reflect the current characteristics of system noise.

Various strategies have been reported to limit the memory of historical information in the filtering process to reduce their contributions while increasing the contributions of the latest information to system state estimation. The fading memory technique uses fading factors to increase the use of latest residuals and reduce the effect of historical information on system state estimation, leading to improved filtering accuracy. It is less expensive in computation and has good numerical stability. B. Kwon studied a fading memory Kalman filter [25]. However, because this method uses the empirical method to determine the fading factor, only suboptimal filtering results can be obtained [26]. As an improvement of the fading memory technique, the limited memory technique can handle the KF divergence caused by model inaccuracy or system noise changes. It uses a limited number of residuals near to the current time to avoid the influence of excessive historical information on system state estimation [27]. Based on the limited memory of residuals, system noise statistics were estimated to improve the KF accuracy [28,29]. However, this method involves a complex update process of state and measurement information, leading to an increased computational load. Wishnef et al. also adopted the limited memory technique to improve KF accuracy, leading to the limited memory-based KF (LM-KF) [30]. Although the limited memory technique reduces the contribution of historical residuals to system state estimation [29,30], since residuals at different epochs within a limited memory are applied with the same weight, it is difficult to distinguish the contributions of residuals at different epochs on system state estimation, leading to a limited improvement of the KF accuracy. Deng et al. also combined the limited memory technique with KF [31]. However, this method determines the initial filtering values based on empiricism. If the initial values are not determined properly, the filtering accuracy will seriously decrease [32]. Yang proposed an adaptive fitting method of system error based on limited memory theory. In this method, the size of system noise statistics is not distinguished, and the system noise and its residual are estimated by the arithmetic mean method (using the same weight) in the selected moving window, which leads to low filtering accuracy [33].

Random-weighted estimation is an advanced statistical calculation method. This method applies random weights to different samples to estimate target parameters. It can achieve unbiased estimation, is simple in computation, and can handle large samples without requiring the accurate distribution of target parameters. It has been used to solve many problems, such as dynamic navigation and positioning [34,35], multi-sensor data fusion [36], M-test in linear models [37], analysis of asymptotic properties of function distribution [38], and estimation of system model error [9,39,40]. Since the application of random weights to different samples provides a solution to address the disadvantage of the limited memory technique due to the use of the same weight to residuals, the combination of the random weighting with limited memory techniques provides a promising solution to estimate unknown system noise statistics for improving KF accuracy.

In this paper, a new random-weighted Kalman filtering method based on limited memory is proposed, and a random-weighted estimation method of system noise statistics is designed. In the moving window, this method adaptively adjusts the weight of noise statistics in limited memory, which suppresses the interference of system noise and excessive historical information on system state estimation, improves the accuracy of filtering calculation, and proves the unbiased nature of random-weighted estimation of system noise statistics. Finally, through simulation and practical experiments, it is proven that the filtering accuracy of the proposed LM-RWKF is much higher than that of KF and LM-KF.

The main contributions of this paper include the following: (i) the random-weighted theories are established based on the concept of limited memory for estimation of system noise statistics; and (ii) a new KF filter is developed by combining the limited memory-based random weighting theories into the KF framework for system state estimation with increased accuracy.

## 3. Computation Scheme

### 3.1. The Concept of Random Weight Estimation

Assume that $x_{1},x_{2},\cdots ,x_{n}$ is a sequence of random variables of independent and identical distribution (iid) with the common distribution function F(x), and its empirical distribution function is defined as follows:

(1) $$F_{n}\left(x\right)=\frac{1}{n}\sum_{i=1}^{N}I_{\left(X_{i}\leq x\right)}$$

where $I_{\left(X_{i\leq }x\right)}$ is the indicator function.

The random weight estimate of $F_{n}\left(x\right)$ can be defined as follows:

(2) $$T_{n}\left(x\right)=\sum_{i=1}^{N}\lambda_{i}I_{\left(X_{i\leq }x\right)}$$

where the random-weighted vector $\left(\lambda_{1},\lambda_{2},\cdots ,\lambda_{n}\right)$ obeys Dirichlet distribution D(1,1,⋅⋅⋅,1), that is, $\sum_{i=1}^{N}\lambda_{i}=1$ and the joint density function of $\left(\lambda_{1},\lambda_{2},\cdots ,\lambda_{n-1}\right)$ is $f(\lambda_{1},\lambda_{2},\cdots ,\lambda_{n-1})=\Gamma (n)$, in which $\Gamma \left(\cdot \right)$ denotes the function, $\left(\lambda_{1},\lambda_{2},\cdots ,\lambda_{n-1}\right)\in D_{n-1}$ and $D_{n-1}=\left\{\left(\lambda_{1},\lambda_{2},\cdots ,\lambda_{n-1}\right):\lambda_{i}\geq 0,i=1,2,\cdots n-1,\;\sum_{i=1}^{N}\lambda_{i}=1\right\}$.

### 3.2. Limited Memory-Based Random-Weighted Estimations of System Noise Statistics

Consider the following dynamic discrete system:

(3) $$\left\{\begin{array}{l} x_{k+1}=\Phi_{k}x_{k}+w_{k}\\ y_{k}=H_{k}x_{k}+v_{k} \end{array}\right.$$

where $x_{k}\in R^{n}$ is the n-dimensional system state vector at time k, $y_{k}\in R^{m}$ the m-dimensional measurement vector, $\Phi_{k}$ the system state transition matrix, $H_{k}$ the system measurement matrix, $w_{k}$ the system process noise, and $v_{k}$ the measurement noise.

Suppose the statistics of system process noise $w_{k}$ are unknown, i.e.,

(4) $$\left\{\begin{array}{l} E\left(w_{k}\right)=a_{k}\\ \;cov(w_{k},\;w_{j})=E\left(w_{k}w_{j}^{T}\right)=A_{k}\delta_{kj} \end{array}\right.$$

where $a_{k}$ and $A_{k}\geq 0$ are the unknown mean and covariance of the process noise, and $\delta_{kj}$ is the Kronecker−δ function.

Suppose the statistical properties of measurement noise $v_{k}$ are unknown, i.e.,

(5) $$\left\{\begin{array}{l} E\left(v_{k}\right)=b_{k}\\ cov(v_{k},\;v_{j})=E\left(v_{k}v_{j}^{T}\right)=B_{k}\delta_{kj} \end{array}\right.$$

where $b_{k}$ and $B_{k}\geq 0$ are the unknown mean and covariance of the measurement noise.

The means of the process and measurement noises are called the first-order noise statistics, while their covariances are called the second-order noise statistics.

In a limited memory period of length N$\left(j=1,2,\cdots ,N\right)$, the arithmetic mean estimation of the measurement noise mean can be expressed as follows:

(6) $${\hat{b}}_{k}=\frac{1}{N}\sum_{j=1}^{N}b_{k-j}$$

Applying the random-weighted concept to Equation (6), the random-weighted estimation of the measurement noise mean is as follows:

(7) $${\hat{b}}_{k}^{*}=\sum_{j=1}^{N}\lambda_{j}b_{k-j}$$

In the limited memory period, the arithmetic mean estimation of the measurement noise covariance can be expressed as follows:

(8) $${\hat{B}}_{k}=\frac{1}{N}\sum_{j=1}^{N}\left[\left(b_{k-j}-{\hat{b}}_{k}\right){\left(b_{k-j}-{\hat{b}}_{k}\right)}^{T}\right]$$

Applying the random-weighted concept to Equation (8), the random weighting estimation of the measurement noise covariance can be obtained as follows:

(9) $${\hat{B}}_{k}^{*}=\sum_{j=1}^{N}\lambda_{j}\left(b_{k-j}-{\hat{b}}_{k}\right){\left(b_{k-j}-{\hat{b}}_{k}\right)}^{T}$$

Define the measurement residual as follows:

(10) $$\beta_{k}=y_{k}-H_{k}{\hat{x}}_{k}$$

According to the first formula in Equation (3), the measurement residual $\beta_{k}$ can be written as follows:

(11) $$\begin{array}{l} \beta_{k}=y_{k}-H_{k}{\hat{x}}_{k}\\ \;\;\;=H_{k}x_{k}+v_{k}-H_{k}{\hat{x}}_{k}\\ \;\;\;=H_{k}\left(x_{k}-{\hat{x}}_{k}\right)+v_{k}\\ \;\;\;=H_{k}{\tilde{x}}_{k}+v_{k} \end{array}$$

where ${\tilde{x}}_{k}=x_{k}-{\hat{x}}_{k}$ denotes the state estimation error.

Similarly, in the limited memory period of length N$\left(j=1,2,\cdots ,N\right)$, the arithmetic mean estimation of the process noise mean can be expressed as follows:

(12) $${\hat{a}}_{k}=\frac{1}{N}\sum_{j=1}^{N}a_{k-j}$$

The arithmetic mean estimation of the process noise covariance can be expressed as follows:

(13) $${\hat{A}}_{k}=\frac{1}{N}\sum_{j=1}^{N}\left[\left(a_{k-j}-{\hat{a}}_{k}\right){\left(a_{k-j}-{\hat{a}}_{k}\right)}^{T}\right]$$

Applying the random-weighted concept to Equations (12) and (13), the random-weighted estimation of $a_{k}$ and $A_{k}$ can be written as follows:

(14) $${\hat{a}}_{k}^{*}=\sum_{j=1}^{N}\lambda_{j}a_{k-j}$$

(15) $${\hat{A}}_{k}^{*}=\sum_{j=1}^{N}\lambda_{j}\left[\left(a_{k-j}-{\hat{a}}_{k}\right){\left(a_{k-j}-{\hat{a}}_{k}\right)}^{T}\right]$$

Define the process residual as follows:

(16) $$\delta_{k}=x_{k+1}-\Phi_{k}{\hat{x}}_{k}$$

According to the second formula in Equation (3), the process residual $\delta_{k}$ can be rewritten as follows:

(17) $$\begin{array}{l} \delta_{k}=x_{k+1}-\Phi_{k}{\hat{x}}_{k}\\ \;\;=(\Phi_{k}x_{k}+w_{k})-\Phi_{k}{\hat{x}}_{k}\\ \;\;=\Phi_{k}\left(x_{k}-{\hat{x}}_{k}\right)+w_{k}\\ \;\;=\Phi_{k}\tilde{x}+w_{k} \end{array}$$

According to the KF principle, we have the following:

(18) $$D_{k}=P_{k}-P_{k\left|k-1\right.}=P_{k}-\Phi_{k}P_{k-1}\Phi_{k}-A_{k}$$

where $D_{k}$ is the process residual covariance, and $P_{k\left|k-1\right.}$ is the one-step state estimation error variance, which is expressed as follows:

(19) $$P_{k\left|k-1\right.}=\Phi_{k}P_{k-1}\Phi_{k}+A_{k-1}$$

$P_{k}$ is the state error covariance, which is expressed as follows:

(20) $$P_{k}=\left(I-K_{k}H_{k}\right)P_{k\left|k-1\right.}{\left(I-K_{k}H_{k}\right)}^{T}+{\hat{B}}_{k}$$

where $K_{k}$ is filter gain matrix, which is expressed as follows:

(21) $$K_{k}=P_{k\left|k-1\right.}H_{k}^{T}{\left(H_{k}P_{k\left|k-1\right.}H_{k}^{T}+{\hat{B}}_{k}\right)}^{-1}$$

Equations (7), (9), (14), and (15) provide the random-weighted estimations of the process noise statistics and measurement noise statistics, which allow us to adaptively adjust the random weights to suppress the interferences of the process and measurement noises on the state estimation for improving the KF accuracy.

### 3.3. Unbiasedness of Random-Weighted Estimations of System Noise Statistics

**Theorem** **1.** *The random-weighted estimations* ${\hat{b}}_{k}^{*}$ *and* ${\hat{a}}_{k}^{*}$ *of* $b_{k}$ *and* $a_{k}$ *, which are given by (7) and (14), are suboptimally unbiased.*

**Proof of Theorem** **1.** From Equation (7), we have the following:

(22) $$\begin{array}{l} E\left({\hat{b}}_{k}^{*}\right)=E\left(\sum_{j=1}^{N}\lambda_{j}b_{k-j}\right)\\ \;\;\;\;=\sum_{j=1}^{N}\lambda_{j}E\left(b_{k-j}\right)\\ \;\;\;\;\neq {\hat{b}}_{k} \end{array}$$

where $\sum_{j=1}^{N}\lambda_{j}=1$ is used in the last step derivation.

It can be seen from Equation (22) that ${\hat{b}}_{k}^{*}$ is not the optimal unbiased estimation of $b_{k}$.

If the measurement noise is constant or involves small variations in the limited memory, i.e., $b_{k}=b_{k-j}$, Equation (22) can be further written as follows:

(23) $$\begin{array}{l} E\left({\hat{b}}_{k}^{*}\right)=\sum_{j=1}^{N}\lambda_{j}E\left(b_{k-j}\right)\\ \;\;\;\;\;\;={\hat{b}}_{k} \end{array}$$

It is known from Equations (22) and (23) that the random weighting estimation ${\hat{b}}_{k}^{*}$ of $b_{k}$ is suboptimally unbiased.

Similarly, from (14), we have the following:

(24) $$\begin{array}{l} E\left({\hat{a}}_{k}^{*}\right)=E\left(\sum_{j=1}^{N}\lambda_{j}a_{k-j}\right)\\ \;\;\;\;\;=\sum_{j=1}^{N}\lambda_{j}E\left(a_{k-j}\right)\\ \;\;\;\;\;\neq a_{k} \end{array}$$

It can be seen from Equation (24) that ${\hat{a}}_{k}^{*}$ is not the optimal unbiased estimation of $a_{k}$.

If the process noise is constant or involves small variations in the limited memory, i.e., $a_{k}=a_{k-j}$, Equation (24) can be further written as follows:

(25) $$\begin{array}{l} E\left({\hat{a}}_{k}^{*}\right)=\sum_{j=1}^{N}\lambda_{j}E\left(a_{k-j}\right)\\ \;\;\;\;=a_{k} \end{array}$$

It is known from Equations (24) and (25) that the random-weighted estimation ${\hat{a}}_{k}^{*}$ of $a_{k}$ is suboptimally unbiased.

The proof of Theorem 1 is completed. □

**Theorem** **2.** *The random-weighted estimations* ${\hat{B}}_{k}^{*}$ *and* ${\hat{A}}_{k}^{*}$ *of* $B_{k}$ *and* $A_{k}$ *, which are given by (9) and (15), are suboptimally unbiased.*

**Proof of Theorem** **2.** Since the state estimate ${\hat{x}}_{k}$ from KF is unbiased, we have the following:

(26) $$\begin{array}{l} E\left({\tilde{x}}_{k}\right)=E\left(x_{k}-{\hat{x}}_{k}\right)\\ \;\;\;=E\left(x_{k}\right)-E\left({\hat{x}}_{k}\right)\\ \;\;\;=0 \end{array}$$

By Equation (11), we have the following:

(27) $$\begin{array}{l} E\left(\beta_{k}\right)=Ε\left(H_{k}{\tilde{x}}_{k}+v_{k}\right)\\ \;\;\;\;=Ε\left(H_{k}{\tilde{x}}_{k}\right)+Ε\left(v_{k}\right)\\ \;\;\;\;=b_{k} \end{array}$$

Calculate the measurement residual covariance:

(28) $$\begin{array}{l} E\left\{\left[\beta_{k}-E(\beta_{k})\right]{\left[\beta_{k}-E(\beta_{k})\;\right]}^{T}\right\}\\ =E\left[(\beta_{k}-b_{k}){\left(\beta_{k}-b_{k}\right)}^{T}\;\right]\\ =E(\beta_{k}\beta_{k}^{T}-\beta_{k}b_{k}^{T}-b_{k}\;\beta_{k}^{T}+b_{k}b_{k}^{T})\\ =E\left[(H_{k}{\tilde{x}}_{k}+v_{k}){\left(H_{k}{\tilde{x}}_{k}+v_{k}\right)}^{T}\right]\\ \;\;\;-E\left[(H_{k}{\tilde{x}}_{k}+v_{k})b_{k}^{T}\right]\;-E\left[b_{k}{\left(H_{k}{\tilde{x}}_{k}+v_{k}\right)}^{T}\right]\\ \;\;\;+E(b_{k}b_{k}^{T})\\ =E(H_{k}{\tilde{x}}_{k}{\tilde{x}}_{k}^{T}H_{k}^{T})+E\left[(v_{k}-b_{k}){\left(v_{k}-b_{k}\right)}^{T}\right]\\ \;\;\;+E(H_{k}{\tilde{x}}_{k}\;\;b_{k}^{T})+E(v_{k}{\tilde{x}}_{k}^{T}H_{k}^{T})-E(H_{k}{\tilde{x}}_{k}\;b_{k}^{T})\\ \;\;\;-E(b_{k}{\tilde{x}}_{k}^{T}H_{k}^{T})\\ =E(H_{k}{\tilde{x}}_{k}{\tilde{x}}_{k}^{T}H_{k}^{T})+B_{k}+E(H_{k}{\tilde{x}}_{k}\;\;v_{k}^{T})\\ \;\;\;\;+E(v_{k}{\tilde{x}}_{k}^{T}H_{k}^{T})-E(B_{k}{\tilde{x}}_{k}\;b_{k}^{T})-E(b_{k}{\tilde{x}}_{k}^{T}H_{k}^{T}) \end{array}$$

where $B_{k}=E\left[(v_{k}-b_{k}){\left(v_{k}-b_{k}\right)}^{T}\right]$.

Since ${\tilde{x}}_{k}$ and $v_{k}$ are independent each other, by Equation (26), we have the following:

(29) $$E(H_{k}{\tilde{x}}_{k}v_{k}^{T})=E(v_{k}{\tilde{x}}_{k}^{T}H_{k}^{T})=0$$

and the following:

(30) $$E(H_{k}{\tilde{x}}_{k}b_{k}^{T})=E(b_{k}{\tilde{x}}_{k}^{T}H_{k}^{T})=0$$

Substituting Equations (29) and (30) into (28) yields the following:

(31) $$\begin{array}{l} E\left[(\beta_{k}-b_{k}){\left(\beta_{k}-b_{k}\right)}^{T}\;\right]=E(H_{k}{\tilde{x}}_{k}{\tilde{x}}_{k}^{T}H_{k}^{T})+B_{k}\\ \;\;\;\;\;\;\;\;\;\;\;\;\;=H_{k}P_{k}H_{k}^{T}+B_{k} \end{array}$$

where $P_{k}=E\left[{\tilde{x}}_{k}\;{\tilde{x}}_{k}^{T}\right]$ is the state error covariance at time k.

From Equation (31), in the limited memory, the arithmetic mean estimation of $B_{k}$ can be calculated as follows:

(32) $$\begin{array}{l} {\hat{B}}_{k}=\frac{1}{N}\sum_{j=1}^{N}B_{k-j}\\ \;=\frac{1}{N}\sum_{j=1}^{N}\left[(\beta_{k-j}-{\hat{b}}_{k-j}){\left(\beta_{k-j}-{\hat{b}}_{k-j}\right)}^{T}-H_{k-j}P_{k-j}H_{k-j}^{T}\right] \end{array}$$

The random-weighted estimation of $B_{k}$ can be written as follows:

(33) $${\hat{B}}_{k}^{*}=\sum_{j=1}^{N}\lambda_{j}\left[(\beta_{k-j}-{\hat{b}}_{k-j}^{*}){\left(\beta_{k-j}-{\hat{b}}_{k-j}^{*}\right)}^{T}-H_{k-j}P_{k-j}H_{k-j}^{T}\right]$$

Taking the mathematical expectation on both sides of Equation (33) generates the following:

(34) $$\begin{array}{l} E\left({\hat{B}}_{k}^{*}\right)=\sum_{j=1}^{N}\lambda_{j}E\left[(\beta_{k-j}-{\hat{b}}_{k-j}^{*}){\left(\beta_{k-j}-{\hat{b}}_{k-j}^{*}\right)}^{T}-H_{k-j}P_{k-j}H_{k-j}^{T}\right]\\ \;\;\;=\sum_{j=1}^{N}\lambda_{j}H_{k-j}P_{k-j}H_{k-j}^{T}+\sum_{j=1}^{M}\lambda_{j}B_{k-j}-\sum_{j=1}^{M}\lambda_{j}H_{k-j}P_{k-j}H_{k-j}^{T}\\ \;\;\;=\sum_{j=1}^{N}\lambda_{j}B_{k-j}\\ \;\;\;\neq B_{k} \end{array}$$

It can be seen from Equation (34) that ${\hat{B}}_{k}^{*}$ is not the optimal unbiased estimation of $B_{k}$.

If the measurement noise is constant or involves small variations in the limited memory period, i.e., $B_{k}=B_{k-j}$, Equation (34) can be further written as follows:

(35) $$\begin{array}{l} E({\hat{B}}_{k}^{*})=\sum_{j=1}^{N}\lambda_{j}B_{k-j}\\ \;\;\;=\sum_{j=1}^{N}\lambda_{j}B_{k}\\ \;\;\;=B_{k} \end{array}$$

It is known from (34) and (35) that the random-weighted estimation ${\hat{B}}_{k}^{*}$ of $B_{k}$ is suboptimally unbiased.

Now let us study the unbiasedness for the random-weighted estimation ${\hat{A}}_{k}^{*}$ of $A_{k}$.

According to Equation (17), we have the following:

(36) $$\begin{array}{l} E\left(\delta_{k}\right)=Ε\left(\Phi_{k}{\tilde{x}}_{k}+w_{k}\right)\\ \;\;\;=Ε\left(\Phi_{k}{\tilde{x}}_{k}\right)+Ε\left(w_{k}\right)\\ \;\;\;=a_{k} \end{array}$$

Calculate the process residual covariance:

(37) $$\begin{array}{l} E\left\{\left[\delta_{k}-E(\delta_{k})\right]{\left[\delta_{k}-E(\delta_{k})\right]}^{T}\right\}\\ \;=E\left[(\delta_{k}-a_{k}){\left(\delta_{k}-a_{k}\right)}^{T}\;\right]\\ \;=E(\delta_{k}\delta_{k}^{T}-\delta_{k}a_{k}^{T}-a_{k}\;\delta_{k}^{T}+a_{k}a_{k}^{T})\\ \;=E\left[(\Phi_{k}{\tilde{x}}_{k}+w_{k}){\left(\Phi_{k}{\tilde{x}}_{k}+w_{k}\right)}^{T}\right]-E\left[(\Phi_{k}{\tilde{x}}_{k}+w_{k})a_{k}^{T}\right]\\ \;\;-E\left[a_{k}{\left(\Phi_{k}{\tilde{x}}_{k}+w_{k}\right)}^{T}\right]+E(a_{k}a_{k}^{T})\\ \;=E(\Phi_{k}{\tilde{x}}_{k}{\tilde{x}}_{k}^{T}\Phi_{k}^{T})+E\left[(w_{k}-a_{k}){\left(w_{k}-a_{k}\right)}^{T}\right]\\ \;+E(\Phi_{k}{\tilde{x}}_{k}\;\;w_{k}^{T})+E(w_{k}{\tilde{x}}_{k}^{T}\Phi_{k}^{T})-E(\Phi_{k}{\tilde{x}}_{k}\;a_{k}^{T})\\ \;\;-E(a_{k}{\tilde{x}}_{k}^{T}\Phi_{k}^{T})\\ \;=E(\Phi_{k}{\tilde{x}}_{k}{\tilde{x}}_{k}^{T}\Phi_{k}^{T})+A_{k}+E(\Phi_{k}{\tilde{x}}_{k}\;\;w_{k}^{T})\\ \;+E(w_{k}{\tilde{x}}_{k}^{T}\Phi_{k}^{T})-E(\Phi_{k}{\tilde{x}}_{k}\;a_{k}^{T})-E(a_{k}{\tilde{x}}_{k}^{T}\Phi_{k}^{T}) \end{array}$$

where $A_{k}=E\left[(w_{k}-a_{k}){\left(w_{k}-a_{k}\right)}^{T}\right]$.

Since ${\tilde{x}}_{k}$ and $w_{k}$ are independent each other, according to Equation (26), we have the following:

(38) $$E(\Phi_{k}{\tilde{x}}_{k}w_{k}^{T})=E(w_{k}{\tilde{x}}_{k}^{T}\Phi_{k}^{T})=0$$

and the following:

(39) $$E(\Phi_{k}{\tilde{x}}_{k}a_{k}^{T})=E(a_{k}{\tilde{x}}_{k}^{T}\Phi_{k}^{T})=0$$

Substituting Equations (38) and (39) into Equation (37) yields the following:

(40) $$\begin{array}{l} E\left[(\delta_{k}-a_{k}){\left(\delta_{k}-a_{k}\right)}^{T}\;\right]=E(\Phi_{k}{\tilde{x}}_{k}{\tilde{x}}_{k}^{T}\Phi_{k}^{T})+A_{k}\\ \;\;\;\;\;\;\;\;\;=\Phi_{k}P_{k}\Phi_{k}^{T}+A_{k} \end{array}$$

where $P_{k}=E\left[{\tilde{x}}_{k}\;{\tilde{x}}_{k}^{T}\right]$ is the state error covariance at time k.

The arithmetic mean estimation of $A_{k}$ can be calculated as

(41) $$\begin{array}{l} {\hat{A}}_{k}=\frac{1}{N}\sum_{j=1}^{N}A_{k-j}\\ \;=\frac{1}{N}\sum_{j=1}^{N}\left[(\delta_{k-j}-{\hat{a}}_{k-j}){\left(\delta_{k-j}-{\hat{a}}_{k-j}\right)}^{T}-\Phi_{k-j}P_{k-j}\Phi_{k-j}^{T}\right] \end{array}$$

By Equation (41), the random-weighted estimation of $A_{k}$ can be written as follows:

(42) $${\hat{A}}_{k}^{*}=\sum_{j=1}^{N}\lambda_{i}\left[(\delta_{k-j}-{\hat{a}}_{k-j}^{*}){\left(\delta_{k-j}-{\hat{a}}_{k-j}^{*}\right)}^{T}-\Phi_{k-j}P_{k-j}\Phi_{k-j}^{T}\right]$$

Taking the mathematical expectation on both sides of Equation (42) generates the following:

(43) $$\begin{array}{l} E\left({\hat{A}}_{k}^{*}\right)=\sum_{j=1}^{N}\lambda_{i}E\left[(\delta_{k-j}-{\hat{a}}_{k-j}^{*}){\left(\delta_{k-j}-{\hat{a}}_{k-j}^{*}\right)}^{T}-\Phi_{k-j}P_{k-j}\Phi_{k-j}^{T}\right]\\ \;\;\;=\sum_{j=1}^{N}\lambda_{j}\Phi_{k-j}P_{k-j}\Phi_{k-j}^{T}+\sum_{j=1}^{M}\lambda_{j}A_{k-j}-\sum_{j=1}^{M}\lambda_{j}\Phi_{k-j}P_{k-j}\Phi_{k-j}^{T}\\ \;\;\;=\sum_{j=1}^{N}\lambda_{j}A_{k-j}\\ \;\;\;\;\neq A_{k} \end{array}$$

It can be seen from Equation (43) that ${\hat{A}}_{k}^{*}$ is not the optimal unbiased estimation of $A_{k}$. 

If the process noise is constant or involves small variations in the limited memory, i.e., $A_{k}=A_{k-j}$, Equation (43) can be further written as follows:

(44) $$\begin{array}{l} E({\hat{A}}_{k}^{*})=\sum_{j=1}^{N}\lambda_{j}A_{k-j}\\ \;\;\;=\sum_{j=1}^{N}\lambda_{j}A_{k}\\ \;\;\;=A_{k} \end{array}$$

It is known from Equations (43) and (44) that ${\hat{A}}_{k}^{*}$ are the random-weighted suboptimal unbiased estimation of $A_{k}$.

The proof of Theorem 2 is completed. □ 

**Figure 1:**
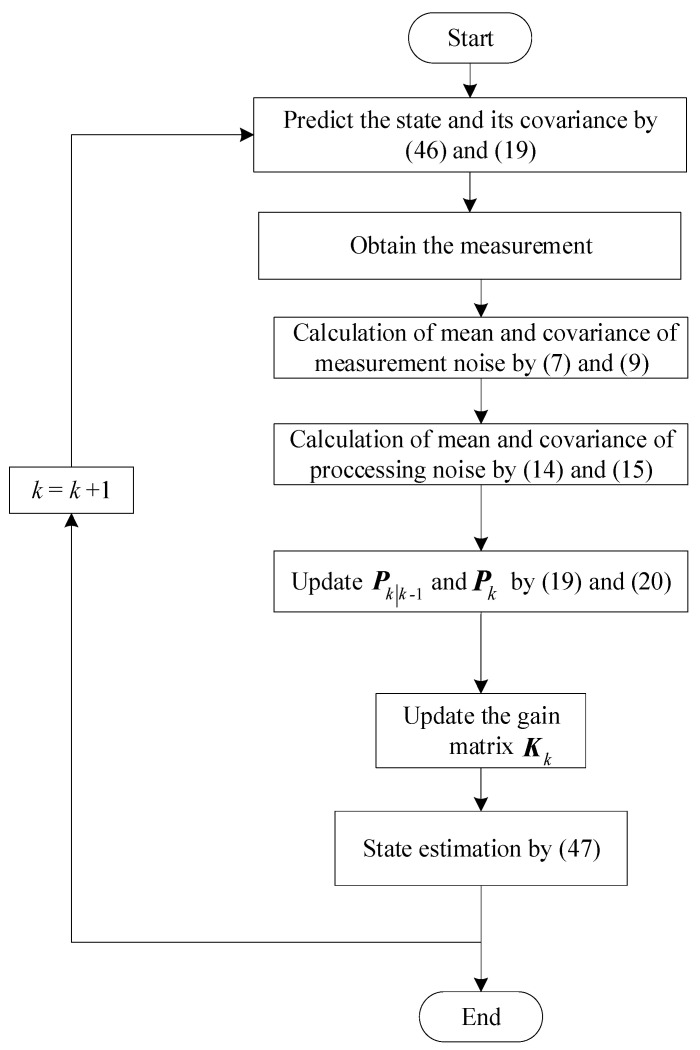
Overview diagram of LM-RWKF.

Based on above, the overview diagram of LM-RWKF is as Figure 1, and the procedure of the proposed LM-RWKF is as follows:

The procedure of the proposed LM-RWKF is as follows:

(i) Initialize the estimated state and its associated error covariance:
  (45) $$\begin{array}{l} {\hat{x}}_{0}=E[x_{0}]\;\\ P_{0}=\mathrm{cov}\left(x_{0},\;x_{0}^{T}\right)=E[(x_{0}-{\hat{x}}_{0}){\left(x_{0}-{\hat{x}}_{0}\right)}^{T}] \end{array}$$
(ii) Calculate predicted state vector:
  (46) $$x_{k|k-1}=\Phi_{k\left|k-1\right.}{\hat{x}}_{k-1}$$
(iii) Calculate the one-step prediction covariance by (19).
(iv) Estimate the mean and covariance of the process and measurement noise statistics by (7), (9), (14), and (15).
(v) The process and measurement noise statistics are fed back to (19)–(21) to obtain a new filter gain matrix.
(vi) Calculate the new state estimation vector.
  (47) $${\hat{x}}_{k}^{*}=x_{k|k-1}^{*}+K_{k}\left(y_{k}-H_{k}x_{k}^{*}-{\hat{b}}_{k}^{*}\right)$$
(vii) Let k = k + 1 return to (ii) until all iterations are complete.

## 4. Performance Evaluation and Discussion

Simulations and experiments were conducted to comprehensively evaluate the performance of the proposed LM-RWKF for dynamic vehicle navigation. The comparison analysis of the proposed LM-RWKF with KF and LM-KF [27] was also conducted to demonstrate the improved performance.

### 4.1. Simulations and Analysis

Computational simulations were conducted to verify the proposed LM-RWKF for tracking the motion of a moving object. The object moves along the three coordinate axes X, Y and Z as per the following equation:

(48) $$\left\{\begin{array}{l} P_{x}=10t+\frac{1}{2}a_{x}t^{2}\\ P_{y}=10t+\frac{1}{2}a_{y}t^{2}\\ P_{z}=10t+\frac{1}{2}a_{z}t^{2} \end{array}\right.$$

where *t* is time, $\left(P_{x},P_{y},P_{z}\right)$ represents the vehicle position, and $\left(a_{x},a_{y},a_{z}\right)$ denotes the vehicle acceleration.

The state vector is as follows:

(49) $$X=\left(P_{x},\;\;\;P_{y},\;\;P_{z}\right)$$

and the processing noise is expressed as follows:

(50) $$w=\left(a_{x},a_{y},a_{z}\right)$$

The moving object is observed in the three axes:

(51) $$\left\{\begin{array}{l} L_{x}=P_{x}+v_{x}\\ L_{y}=P_{y}+v_{y}\\ L_{z}=P_{z}+v_{z} \end{array}\right.$$

where $\left(L_{x},L_{y},L_{z}\right)$ represents the measurement vector, and $\left(v_{x},v_{y},v_{z}\right)$ denotes the measurement noise. 

**Figure 2:**
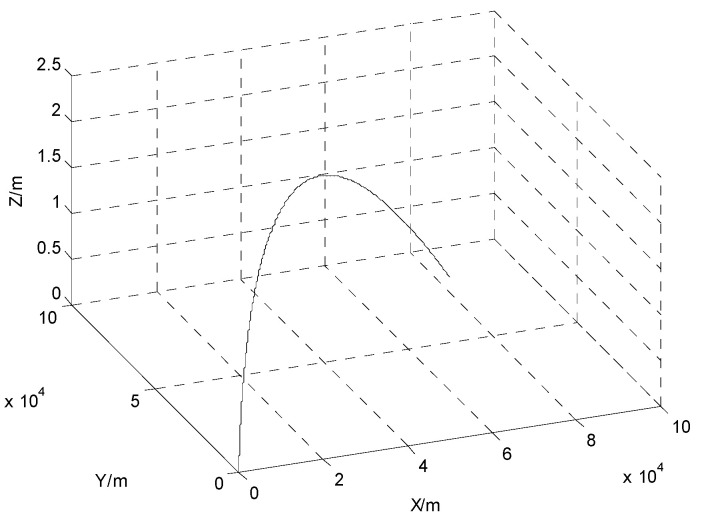
Trajectory of the moving object.

The simulation time was 1200 s and the sampling cycle was 1 s. The limited memory length was set to *N* = 20. The trajectory of the moving object is shown in Figure 2. The initial parameters are given in Table 1.

#### 4.1.1. Estimation of System Noise Statistics

Simulation trials were conducted to examine the accuracy of LM–RWKF for estimation of the measurement noise statistics. The initial state and its estimate were set as follows:

(52) $$x_{0}=\left[\begin{array}{ccc} 0.1 & 0.1 & 0.1 \end{array}\right],\;{\hat{x}}_{0}=\left[\begin{array}{ccc} 0.1 & 0.1 & 0.1 \end{array}\right]\;$$

The initial estimation error covariance was chosen as follows:

(53) $${\hat{P}}_{0}=\left[\begin{array}{ccc} 1 & 0 & 0\\ 0 & 1 & 0\\ 0 & 0 & 1 \end{array}\right]$$

The true values and initial estimates of the measurement noise mean and covariance were as follows:

(54) $$b_{k}=\left[1,\;\;1,\;\;1\right],B_{k}\;=\left[\begin{array}{ccc} 15 & 0 & 0\\ 0 & 15 & 0\\ 0 & 0 & 15 \end{array}\right]$$

(55) $${\hat{b}}_{0}=\left[\begin{array}{ccc} 0.2 & 0.2 & 0.2 \end{array}\right],{\hat{B}}_{0}\;=\left[\begin{array}{ccc} 0.2 & 0 & 0\\ 0 & 0.2 & 0\\ 0 & 0 & 0.2 \end{array}\right]$$

The true values and initial estimates of the process noise mean and covariance were as follows:

(56) $$a_{k}=\left[0,0,0\right],A_{k}\;=\left[\begin{array}{ccc} 3 & 0 & 0\\ 0 & 3 & 0\\ 0 & 0 & 3 \end{array}\right]$$

(57) $${\hat{a}}_{0}=\left[0,0,0\right],\;{\hat{A}}_{0}=\left[\begin{array}{ccc} 1 & 0 & 0\\ 0 & 1 & 0\\ 0 & 0 & 1 \end{array}\right]$$

**Figure 3:**
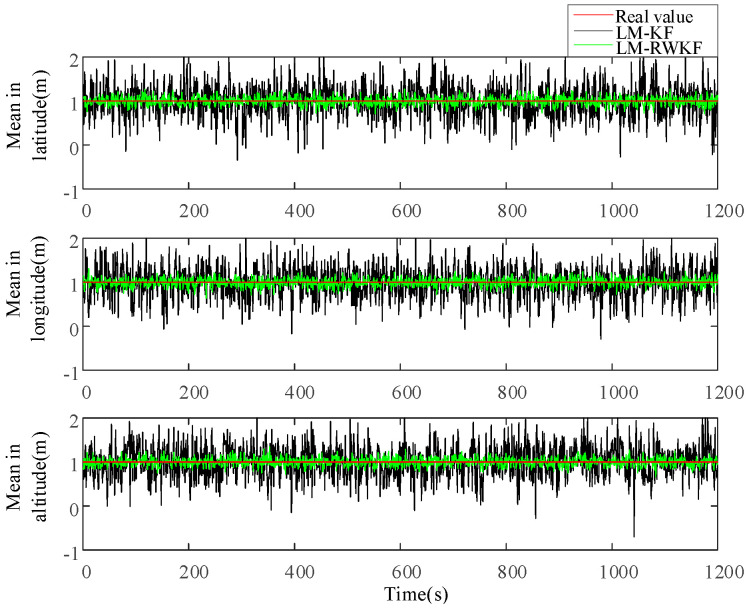
Estimations of the measurement noise mean by LM–KF and LM–RWKF.

**Figure 4:**
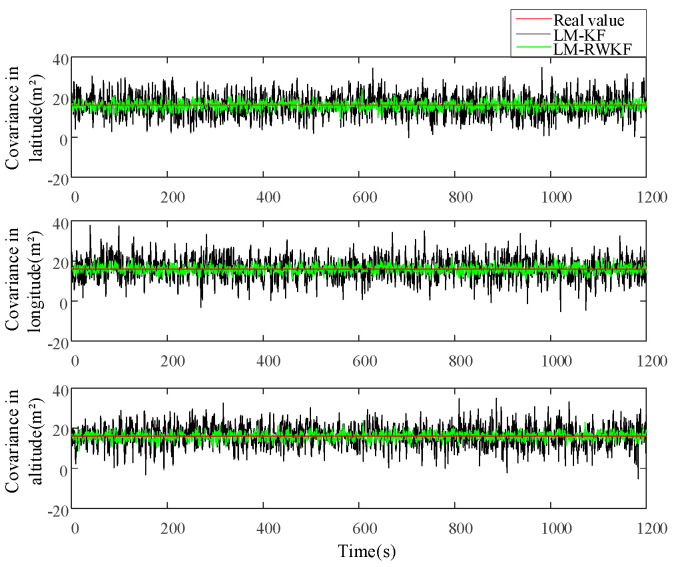
Estimations of the measurement noise covariance by LM–KF and LM–RWKF.

Figure 3 and Figure 4 illustrate the measurement noise means and covariances estimated by both LM–KF and LM–RWKF, and their mean absolute errors (MAEs) are listed in Table 2. As shown in Figure 3, the LM–KF estimation curve of the measurement noise mean involves large-magnitude oscillations, leading to the 0.564 m MAE. By contrary, the oscillations involved in the LM–RWKF estimation curve are much smaller in magnitude than those in the LM–KF estimation curve, leading to the 0.161 m MAE. As shown in Figure 4, both LM–KF and LM–RWKF estimations of the measurement noise covariance exhibit a similar trend in the case of the measurement noise mean estimation. The MAE of the measurement noise covariance estimated by LM–KF is 8.137 $m^{2}$, while the MAE by LM–RWKF is 1.061 $m^{2}$, which is very close to the true value.

**Figure 5:**
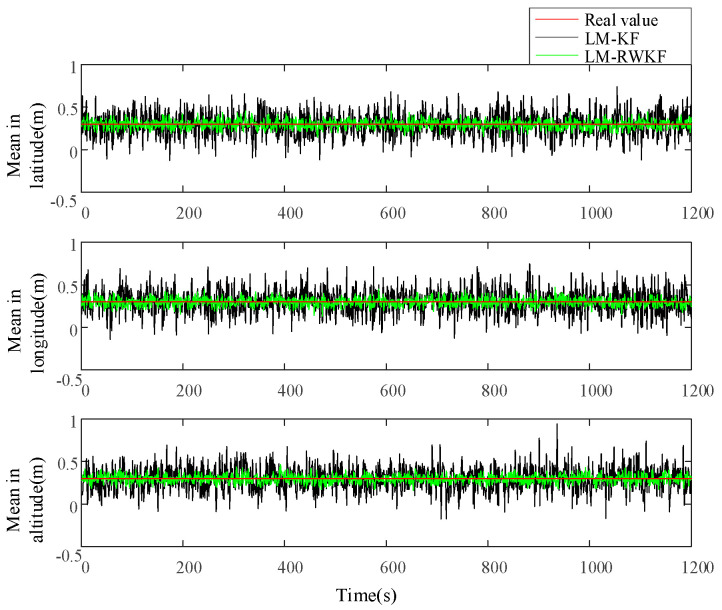
Estimations of the process noise mean by LM–KF and LM–RWKF.

**Figure 6:**
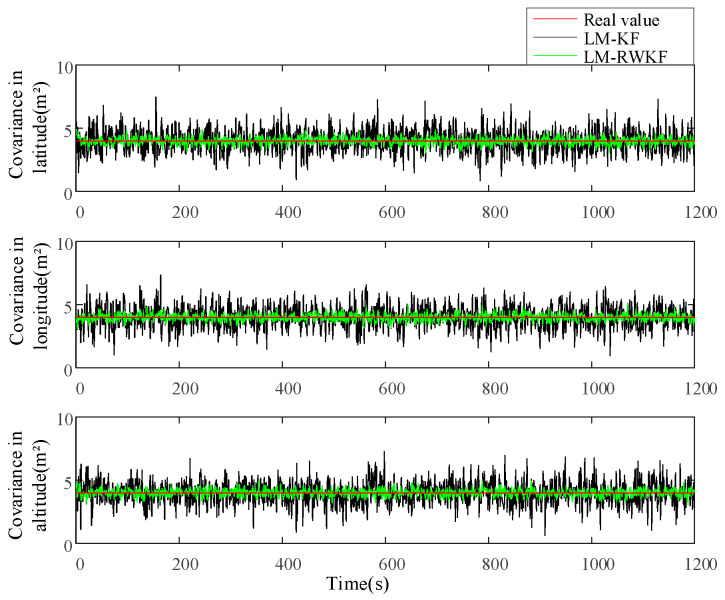
Estimations of the process noise covariance by LM–KF and LM–RWKF.

Figure 5 and Figure 6 show the process noise means and covariances estimated by both LM–KF and LM-RWKF, while their MAEs are listed in Table 3. As shown in Figure 5, the LM–KF estimation curve of the process noise mean involves large-magnitude oscillations, leading to the MAE of 0.122 m. By contrast, the oscillations involved in the LM–RWKF estimation curve are much smaller in magnitude than those in the LM–KF estimation curve, leading to the MAE of 0.057 m. As shown in Figure 6, the LM–KF estimation of the process noise covariance involves large-magnitude fluctuations, leading to the MAE of 1.114 $m^{2}$. Nevertheless, the MAE of the process noise covariance estimated by LM–RWKF is 0.369 $m^{2}$, much smaller than that of LM-KF.

The above results demonstrate that LM–RWKF can effectively estimate system noise statistics, leading to higher estimation accuracy than LM–KF.

#### 4.1.2. Vehicle Position Estimation

**Figure 7:**
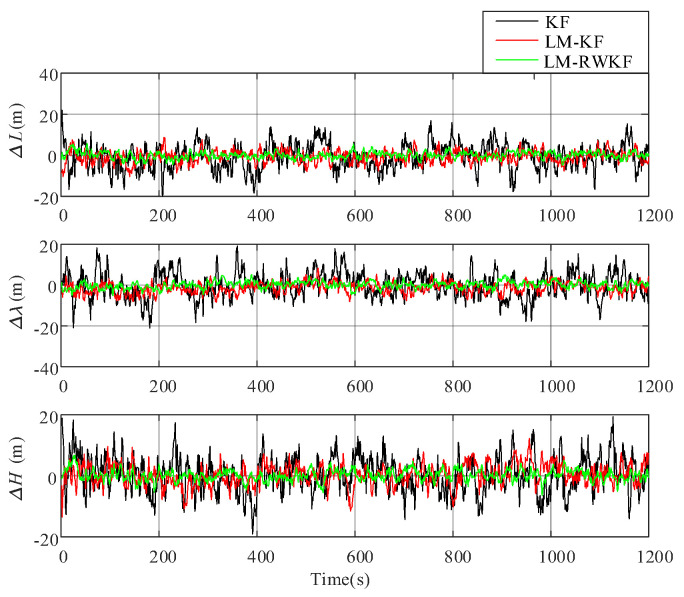
Moving object’s position errors estimated by KF, LM–KF and LM–RWKF.

The position error of the moving vehicle is also estimated under the same conditions by both KF, LM–KF, and LM–RWKF. Figure 7 shows the position error curves of KF, LM–KF, and LM–RWKF. Table 4 also provides the position MAEs of these three methods. As shown in Figure 7, the KF estimation curve involves large-magnitude oscillations, leading to the 8.515 m MAE. The oscillation magnitude is decreased by LM–KF due to its capability of noise statistics estimation. However, since it applies the same weight to the noise statistics within a limited memory, the LM–KF improvement is still limited, leading to the 4.953 m MAE. By contrast, since LM–RWKF adopts the RW estimation of system noise statistics, its mean absolute error in position is 1.235 m, which is much smaller than those of KF and LM–KF.

#### 4.1.3. Computational Load Analysis

**Figure 8:**
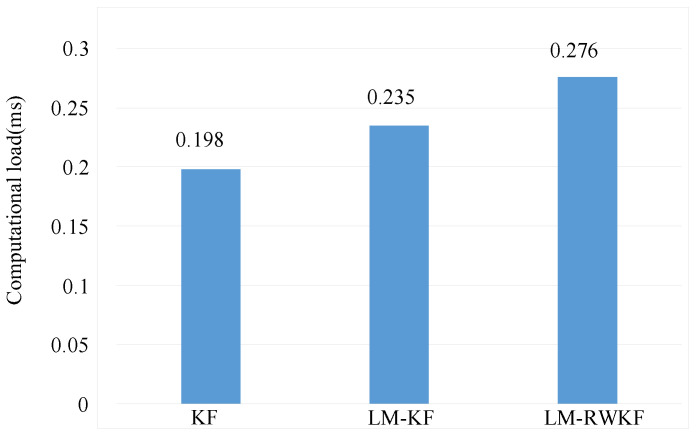
Comparison of computational load.

The computational performances of KF, LM–KF, and LM–RWKF were studied based on the above simulations, which were implemented on a PC (Intel^®^ Core™ i5 12100F CPU, Intel, Santa Clara, CA, USA). As shown in Figure 8, KF has the smallest computational time, leading to a mean of 0198 ms per iteration. Due to the involvement of the noise estimation, the mean computational time of LM–KF is 0.235 ms per iteration, which is larger than that of KF. Since the random weight principle is further involved, the mean computational time of LM–RWKF is 0.276 ms per iteration. However, similar to KF, the computational time of LM–RWKF is much above the threshold of 0.07 s per iteration for real-time performance.

In practical engineering, the size of limited memory window is closely related to the filtering accuracy. The longer the limited memory window is, the higher the filtering precision and the larger the computational load will be. Therefore, the practical application should select an appropriate length of the limited memory window to balance the filtering accuracy and the calculation amount.

### 4.2. Experiments and Analysis

Experiments were also conducted to evaluate the performance of LM-RWKF in comparison with LM-KF and KF for navigation of a ground vehicle using a BDS/MEMS IMU (Bei Dou Satellite Navigation System/Micro-Electro-Mechanical System Inertial Measurement Unit) integrated navigation system.

#### 4.2.1. BDS/SINS Integrated Navigation System Mathematical Model

The state vector of the BDS/MEMS IMU integrated navigation system is defined as follows:

(58) $$X\left(t\right)={\left[\delta v_{E},\delta v_{N},\delta v_{U},\delta L,\delta h,\delta \lambda ,\;\varepsilon_{x},\;\varepsilon_{y},\;\varepsilon_{z},\nabla_{x},\nabla_{y},\nabla_{z}\right]}^{T}$$

where $\left(\delta v_{E},\delta v_{N},\delta v_{U}\;\right)$ are the velocity errors of the aircraft in East, North, and Up, (δL,   δλ,   δh) are the errors in latitude, longitude, and altitude, ($\varepsilon_{x},\;\varepsilon_{y},\;\varepsilon_{z}$) is the constant drift of the gyro, and ($\nabla_{x},\;\nabla_{y},\;\nabla_{z}$) is the zero bias of the accelerometer.

The system state equation of the BDS/ MEMS IMU integrated navigation is described by the following:

(59) $$\dot{X}\left(t\right)=F\left(t\right)X\left(t\right)+G(t)w(t)$$

where $F\left(t\right)$ is the system function, and w(t) is the system process noise consisting of the gyro’s Gaussian white noise $\left(w_{gx},\;w_{gy},\;w_{gz}\right)$ and accelerometer’s Gaussian white noise $\left(w_{ax},\;w_{ay},\;w_{az}\right)$, i.e.,

(60) $$w(t)={\left[w_{gx},w_{gy},w_{gz},w_{ax},w_{ay},w_{az}\right]}_{6\times 1}^{T}$$

$G\left(t\right)$ is the coefficient matrix of the system noise and is expressed as follows:

(61) $$G(t)={\left[\begin{array}{cc} C_{b}^{n} & 0_{3\times 3}\\ 0_{3\times 3} & C_{b}^{n}\\ 0_{9\times 3} & 0_{9\times 3} \end{array}\right]}_{15\times 6}$$

where $C_{b}^{n}$ is the conversion matrix from the body coordinate system to navigation coordinate system.

The measurement equation of the BDS/MEMS IMU integrated navigation system is established using the velocity error and position error as measurement information.

The measurement equation of position error is described by the following:

(62) $$Z_{p}\left(t\right)=H_{p}\left(t\right)X\left(t\right)+V_{p}\left(t\right)=\left[\begin{array}{c} R\cos L\delta \lambda +n_{E}\\ R\delta L+n_{N}\\ \delta h+n_{U} \end{array}\right]$$

where $H_{p}\left(t\right)$ is the position measurement matrix, which is expressed as follows:

(63) $$H_{p}={\left[0_{3\times 3}\;\vdots \;diag\left[R\;R\cos L\;1\right]\;\vdots \;0_{3\times 9}\right]}_{9\times 15}$$

$V_{p}\left(t\right)$ is the position measurement noise, which is expressed as follows:

(64) $$V_{p}\left(t\right)={\left[\begin{array}{ccc} n_{E} & n_{N} & n_{U} \end{array}\right]}^{T}$$

where $n_{E}$, $n_{N}$, and $n_{U}$ are the position errors of the BDS receiver in the three axes, respectively.

The velocity error measurement equation can be written as follows:

(65) $$Z_{v}\left(t\right)=H_{v}\left(t\right)X\left(t\right)+V_{v}\left(t\right)=\left[\begin{array}{c} \delta v_{E}+n_{vE}\\ \delta v_{N}+n_{vN}\\ \delta v_{U}+n_{vU} \end{array}\right]$$

where $H_{v}\left(t\right)$ is the velocity measurement matrix, which is expressed as follows:

(66) $$H_{v}\left(t\right)={\left[diag\left[1\;1\;1\right]\vdots \;0_{3\times 12}\right]}_{9\times 15}$$

$V_{v}\left(t\right)$ is the velocity measurement noise, which is expressed as follows:

(67) $$V_{v}\left(t\right)={\left[\begin{array}{ccc} n_{v_{E}} & n_{v_{N}} & n_{v_{U}} \end{array}\right]}^{T}$$

where $n_{v_{E}}$, $n_{v_{N}}$, and $n_{v_{U}}$ are the velocity measurement errors of the BDS receiver in the three axes, respectively.

According to Equations (62) and (65), the measurement equation of the BDS/MEMS IMU integrated navigation system can be obtained as follows:

(68) $$Z\left(t\right)=\left[\begin{array}{c} H_{p}\left(t\right)\\ H_{v}\left(t\right) \end{array}\right]X\left(t\right)+\left[\begin{array}{c} V_{p}\left(t\right)\\ V_{v}\left(t\right) \end{array}\right]=H\left(t\right)X\left(t\right)+V\left(t\right)$$

#### 4.2.2. Experimental Setup

**Figure 9:**
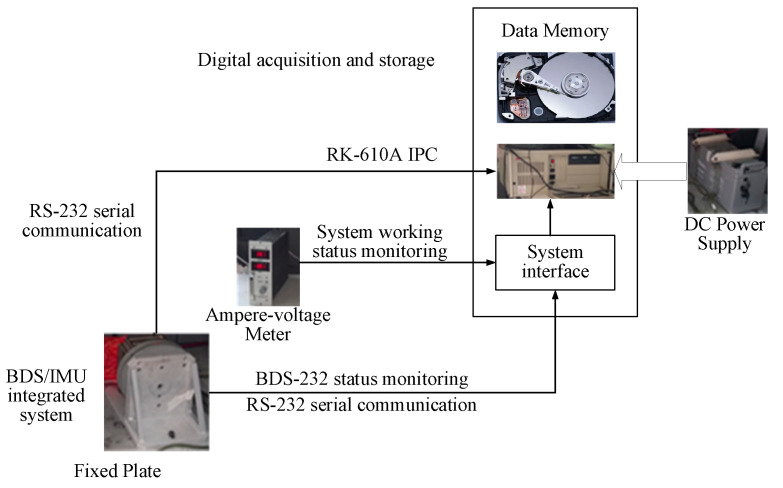
The framework of the experimental system.

Practical experiments were also conducted to evaluate the performance of the proposed improved LM-RWKF algorithm for vehicle navigation. The vehicle used s BDS/ MEMS IMU integrated system for navigation. The BDS/MEMS IMU integrated navigation system adopted the East-North-Up geography frame as the navigation frame. The test vehicle is a gray Mazda SUV. The BDS/MEMS IMU integrated navigation system is installed on the Mazda off-road vehicle. The BDS/MEMS IMU integrated navigation system includes a set of IMU and Bei Dou receivers with NV-IMU300 model, and the BeiDou receiver outputs C/A code measurement at a data update rate of 1 Hz. The test vehicle also carries related auxiliary equipment, including a DC power supply, small computer, data processor, and voltmeter. Figure 9 shows the structure of the test system.

The parameters of the BDS/MEMS IMU integrated navigation system are provided in Table 5. The initial flight parameters of the UAV are shown in Table 6. The test time for the filtering calculation was 1200 s. The filtering time step was 0.1 s.

**Figure 10:**
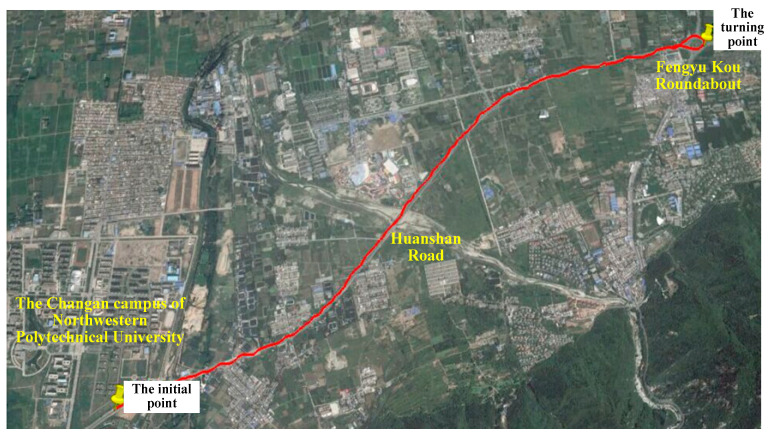
Vehicle traveling trajectory.

**Figure 11:**
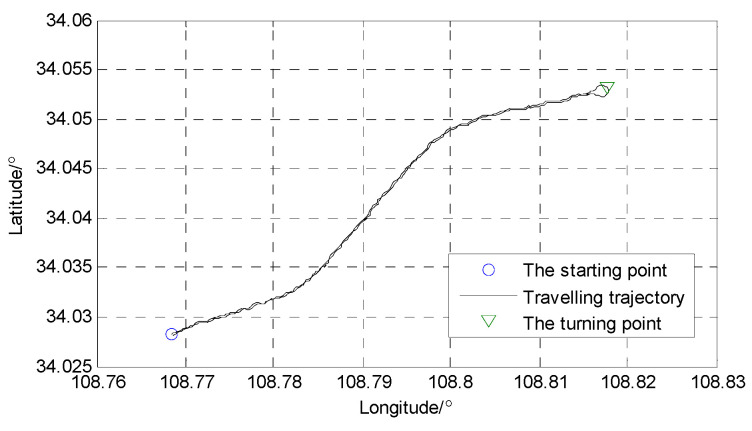
The position coordinates of the vehicle travelling trajectory.

The vehicle was traveling along South Qinling North Road in Xi’an, with the initial starting position being 34°01′41.24″ North latitude and 108°46′05.89″ east longitude. After arriving at Qinling Ring Island on Huanshan Road, it turned at 34°03′10.28″ N, 108°49′04.61″ E and returned to the initial position. The driving route and location of the experimental vehicle are shown in Figure 10 and Figure 11. The driving distance is 15.38 km, the driving time is 20 min, and the average speed of the vehicle is 35 km/h. When the vehicle is moving, the Beidou receiver receives more than seven satellite signals and uses the information obtained by the differential Beidou system as reference data for positioning errors.

#### 4.2.3. Experimental Results and Analysis

**Figure 12:**
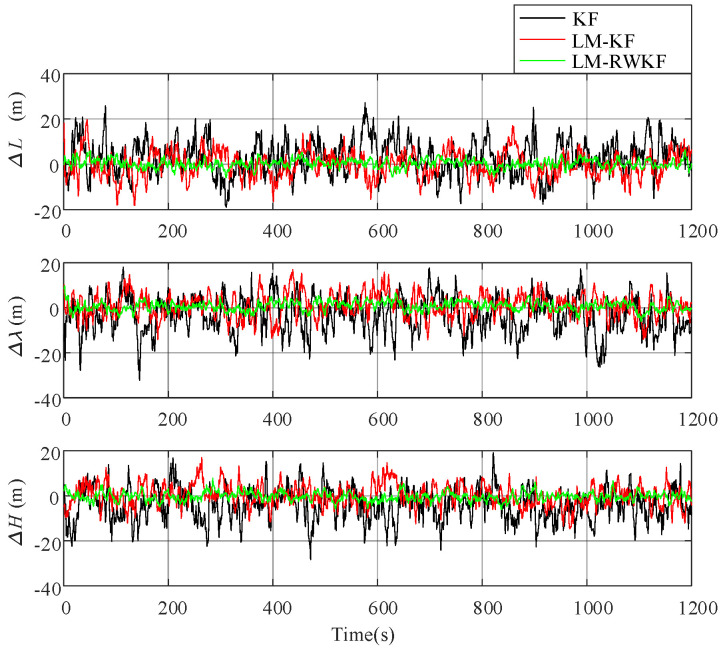
Position errors of KF, LM–KF, and LM–RWKF.

For the purpose of comparison analysis, trials were conducted by using KF, LM–KF, and LM–RWKF, respectively. Figure 12 shows the position errors of these three methods, and Table 5 provides their MAEs.

As shown in Table 7 and Figure 10, KF has large-magnitude oscillations in the filtering curve, and its position MAE is 13.612 m. Although LM–KF improves KF, leading to the decreased oscillation magnitude, its improvement is still limited, resulting in the 8.587 m position MAE. In contrast, LM–RWKF significantly decreases the oscillation magnitude, and its position MAE is 2.421 m, which is much smaller than those of the other two methods.

## 5. Conclusions

This paper proposes a new LM-RWKF for system state estimation in the presence of unknown or inaccurate system noise statistics by combining the random weighting concept with limited memory technique. Random-weighted theories are established based on the limited memory technique to estimate system noise statistics within a limited memory, which are further fed back to KF for system state estimation. The proposed method cannot only adaptively adjust the weights to suppress the interference of noise statistics on system state estimation, but it can also suppress the influence of too much historical information on system state estimation, since the random weighting estimations of system noise statistics are established within a limited memory and embedded with random weights. Simulations, experiments, and comparison analysis demonstrate that the proposed LM-RWKF can effectively estimate system noise statistics, leading to higher accuracy than LM-KF in the presence of unknown or biased noise statistics.

In the existing methods, the length of the moving window is selected according to experience. Future work needs to study how to accurately select the window length, as well as the relationship between the length of the moving window and the filtering calculation accuracy, in order to find a balance between the length of the moving window, the filtering accuracy, and the computational workload. In addition, we will investigate how to combine the proposed improved LM-RWKF algorithm with the concept of artificial intelligence to develop an intelligent filtering algorithm that can accurately estimate sensor errors and system noise statistics.

## Figures and Tables

**Table 1:** Initial parameters.

Parameters		Value
Initial position	East longitude	108.736°
	North latitude	34.246°
	Altitude	3500 m
Initial velocity	East	0 m/s
	North	100 m/s
	Up	0 m/s
Initial attitude	Pitch	0°
	Roll	0°
	Yaw	0°
Initial position error	East longitude	10 m
	North latitude	10 m
	Altitude	10 m
Initial velocity error	East	0.5 m/s
	North	0.5 m/s
	Up	0.5 m/s
Initial attitude error	Pitch	1′
	Roll	1′
	Yaw	1.5′
Initial variances	Position	0.2 m
	Velocity	$9.0\times 10^{-6}m^{2}s^{-2}$

**Table 2:** MAEs of the system noise statistics estimated by LM–KF and LM–RWKF.

**Filtering Methods**	**Measurement Noise Mean**	**Measurement Noise Covariance**
LM–KF	0.564 m	$8.137\;m^{2}$
LM–RWKF	0.161 m	$1.061\;m^{2}$

**Table 4:** Moving object’s position MAEs by KF, LM–KF and LM–RWKF.

Filtering Methods	MAE
KF	8.515 m
LM–KF	4.953 m
LM–RWKF	1.235 m

**Table 5:** The parameters of the BDS / MEMS IMU integrated navigation system.

Sensors	Error Sources	Values (1σ)
IMU	Gyro constant drift	0.1°/h
	Gyro white noise	0.05°/h
	Accelerometer zero bias	$1\;\times \;10^{-3}$ g
	Accelerometer white noise	$1\;\times \;10^{-4}$ g
BDS receiver	Data update rate	1 Hz
	Positioning accuracy	15 m
	Velocity accuracy	0.05 m/s

**Table 6:** Initial position parameters.

Parameters		Value
Initial position	East longitude	108°46′05.89″
	North latitude	34°01′41.24″
	Altitude	2 m
Initial velocity	East	0 m/s
	North	120 m/s
	Up	0 m/s
Initial position error	East longitude	10 m
	North latitude	10 m
	Altitude	12 m
Initial velocity error	East	0.5 m/s
	North	0.5 m/s
	Up	0.5 m/s

**Table 7:** MAEs by KF, LM–KF, and LM–RWK for the UAV position.

Filtering Methods	MAE
KF	13.612 m
LM–KF	8.587 m
LM–RWKF	2.421 m

**Table 3:** MAEs of the process noise statistics estimated by LM–KF and LM–RWKF.

Filtering Methods	Measurement Noise Mean	Measurement Noise Covariance
LM–KF	0.122 m	$1.114\;m^{2}$
LM–RWKF	0.057 m	$0.369\;m^{2}$

## Data Availability

Data are contained within the article.
